# FSim: A Novel Functional Similarity Search Algorithm and Tool for Discovering Functionally Related Gene Products

**DOI:** 10.1155/2014/509149

**Published:** 2014-08-12

**Authors:** Qiang Hu, ZhiGang Wang, ZhengGuo Zhang

**Affiliations:** Department of Biomedical Engineering, Institute of Basic Medical Sciences, Chinese Academy of Medical Sciences, School of Basic Medicine, Peking Union Medical College, Beijing 100005, China

## Abstract

*Background*. During the analysis of genomics data, it is often required to quantify the functional similarity of genes and their products based on the annotation information from gene ontology (GO) with hierarchical structure. A flexible and user-friendly way to estimate the functional similarity of genes utilizing GO annotation is therefore highly desired. *Results*. We proposed a novel algorithm using a level coefficient-weighted model to measure the functional similarity of gene products based on multiple ontologies of hierarchical GO annotations. The performance of our algorithm was evaluated and found to be superior to the other tested methods. We implemented the proposed algorithm in a software package, FSim, based on *R* statistical and computing environment. It can be used to discover functionally related genes for a given gene, group of genes, or set of function terms. *Conclusions*. FSim is a flexible tool to analyze functional gene groups based on the GO annotation databases.

## 1. Introduction

In genomic studies, utilizing high-throughput techniques, such as high-density microarrays and next-generation sequencing, researchers can often identify a set of candidate genes from the large amount of data. A common task is to discover the functional relationships of the identified set of genes. Several classic approaches have been developed to compare the functional relationships among gene products. Sequence alignment is one of these classical methods to search the homology of gene products. However, many gene products with homologous sequences are not functionally similar and vice versa. Coexpression data, derived from microarray experiments for example, are usually carried out to discover disease-related genes—based on the assumption that the coregulated genes from expression data are considered functionally related. Similar to sequence homology, genes with coexpression pattern are not necessarily functionally related and vice versa. These limitations thus call for the development of alternative approaches to measure the similarities among gene products.

Semantic similarity analysis based on the information from annotation databases of gene products is a straightforward and efficient way to quantify functional similarity. A number of comprehensive databases, such as Gene Ontology (GO) [1], Kyto Encyclopedia of Genes and Genomes (KEGG) [2], Reactome pathways [3], Swiss-Prot [4, 5], SMART Domains [6], and the NCBI Online Mendelian Inheritance in Man (OMIM) [7], are available to provide functional annotation information for gene products. Among them, GO terms have been widely used to annotate the functions of gene products [3, 8, 9]. The GO database provides a controlled vocabulary of terms to describe gene product functions. Specifically, three hierarchical structured vocabularies (ontologies) were developed to describe gene products in terms of their associated biological processes (BP), cellular components (CC), and molecular functions (MF).

The rich annotation information from the GO database has been adopted by many algorithms to calculate the semantic similarity between genes [10–12], and various tools based on these algorithms have been developed. For example, GOTax [13] provides a functional similarity search tool for a group of proteins and protein families, with their GO annotations as input. G-SESAME [14] is an online tool for measuring the semantic similarity between two genes. GOSim [15] is an *R* package integrating multiple functional similarity algorithms based on information content (IC) of GO terms. FunSimMat [16, 17] is an online database offering various precomputed functional similarity values by IC-based methods for proteins in UniProtKB and for protein families in Pfam and SMART. DAVID [18] database provided a gene functional classification tool according to the functional dissimilarities with multiple annotation databases including GO. These methods performed well when the relationship between two gene products was measured within one of the three GO ontologies. However, using a single ontology only will likely miss important information stored in other ontologies and result in lower sensitivity. It is thus highly desirable to develop methods to measure the functional relationships of large amount of gene products using multiple ontologies.

Here, we propose a novel algorithm to measure the functional similarity of gene products using multiple ontologies within the hierarchical GO framework. We have developed a package based on the *R* environment, called FSim, to search functionally related genes by a given gene, a group of GO terms, a set of genes, or even biological keywords. The performance of this package was compared with publically available representative methods and found to outperform these methods.

## 2. The Existing Methods

### 2.1. The Kappa Method

The kappa statistical measure of cooccurrence between two sets of categorized data is adopted in the DAVID database [18]. For two given GO annotation of genes *m* and *n*, *O*_*mn*_ represents the observed cooccurrence and *A*_*mn*_ represents chance cooccurrence. The kappa value between genes *m* and *n* is defined as follows:

(1) $$\begin{array}{c} K_{mn}=\frac{O_{mn}-A_{mn}}{1-A_{mn}}. \end{array}$$

The DAVID tool integrates multiple annotation categories including GO, KEGG pathways, and BioCarta pathways. Most of the annotation sources do not have hierarchical structures like GO, so the tool uses the kappa method directly on the equally weighted flat matrix. In other words, DAVID treats the GO annotation as an unweighted flat matrix.

### 2.2. The IC Based Methods

Information content (IC) is a concept taken from information theory, which refers to the negative logarithmic value of the probability of an object. The IC value represents the amount of information of the object, so it can be used to calculate the specificity of GO terms. Most of the existing algorithms that measure the functional similarities of gene products are based on the IC values of annotated GO terms. These methods include three primary steps to calculate the similar scores. First, the IC values of all GO terms in the annotation database are calculated according to their probability values:

(2) $$\begin{array}{c} \text{IC}\left(t_{i}\right)=-\log P\left(t_{i}\right), \end{array}$$

where *t*_*i*_ is the *i* GO term in the annotation database.

Second, the semantic similarity between each two GO terms is measured. For example, Lin's pairwise similarity between GO terms (*t*_*i*_ and *t*_*j*_) is defined as

(3) $$\begin{array}{c} {\text{sim}}_{\text{Lin}}\left(t_{i},t_{j}\right)=\frac{2{\text{IC}}_{ms}\left(t_{i},t_{j}\right)}{\text{IC}\left(t_{i}\right)+\text{IC}\left(t_{j}\right)}, \end{array}$$

where IC_*ms*_(*t*_*i*_, *t*_*j*_) denotes the information content of the minimums subsumer of the two terms, which is the lowest common ancestor in the GO hierarchy.

Schlicker et al. [19] proposed a relevance method based on Lin's method:

(4) $$\begin{array}{c} {\text{sim}}_{\text{Relevance}}\left(t_{i},t_{j}\right)={\text{sim}}_{\text{Lin}}\left(t_{i},t_{j}\right)\times \left(1-e^{-{\text{IC}}_{ms}(t_{i},t_{j})}\right), \end{array}$$

where sim_Lin_ is Lin's similarity method to compare GO terms *t*_*i*_ and *t*_*j*_ and *e* is the mathematical constant.

Third, functional similarity methods (funSimMax), such as BMA (best match average) [20] and Hausdorff distances, are adopted to measure the functional similarity between genes with different groups of GO terms. Considering that two genes (*g* and *g'*) are annotated with two groups of GO terms with lengths *M* and *N*, respectively, a similarity matrix can be calculated between terms from the two groups. The funSimMax method is defined as the average of the column and row maximum scores:

(5) SimfunSimMax(g,g′) =mean⁡{1N∑i=1Nmax⁡1≤j≤M⁡(sim(ti,tj)),1M∑i=1Mmax⁡1≤j≤N⁡(sim(ti,tj))},

where sim is the semantic method to compare the similarity of GO terms *t*_*i*_ and *t*_*j*_. *M* and *N* are the lengths of GO terms for gene *g* and gene *g'*, respectively.

del Pozo et al. [21] proposed a method (Hausdorff) to calculate the functional distance according to the Hausdorff matrices. Given gene *g* and gene *g'* with GO term sets *A* and *B*, the Hausdorff distance from set *A* to *B* is

(6) $$\begin{array}{c} D_{\text{hausdorff}}^{a\to b}=\underset{a\in A}{\max}\left\{\underset{b\in B}{\min}\left(\text{sim}\left(a,b\right)\right)\right\}, \end{array}$$

where sim is the semantic method to compare the similarity of GO term *a* and term *b*. As the Hausdorff distance is not symmetrical, the similarity distance between *g* and gene *g'* is defined as

(7) $$\begin{array}{c} {\text{Sim}}_{\text{Hausdorff}}=\max\left(D_{\text{hausdorff}}^{a\to b},D_{\text{hausdorff}}^{b\to a}\right). \end{array}$$

In this paper, we compared the performances of these IC based methods with that of our proposed approach.

## 3. The Proposed FSim Algorithm

### 3.1. Data Sources

The annotation packages “GO.db,” “org.Hs.eg.db,” and “KEGG.db” from the bioconductor project [22] were used to compute the similarity scores of gene products. All these data packages were built from the current release of the GO database, including 43059 annotated human genes and 36376 GO terms. These include 23878 for BP ontology, 3045 for CC ontology, and 9453 for MF ontology, separately. Validation data sets were built from the KEGG database. Six of the 223 curated human pathways in the KEGG database have less than 5 genes and are filtered out. The remaining 223 human pathways were then used to generate a validation data set for the evaluation with different algorithms.

### 3.2. Level Coefficient (LC)

We define a variant, level coefficient (LC), to describe the weight of the information contained with a GO term. GO terms are structured as a directed acyclic graph (DAG), constructing a hierarchical tree with terms as nodes and leaves and connections between terms as edges (see Supplementary Figure 1 in Supplementary Material available online at http://dx.doi.org/10.1155/2014/509149). Two related terms are connected with an edge denoting the parent-child relationship between nodes. Parent refers to the node closer to the root(s) of the graph and child to that closer to the leaf nodes. GO terms are structured in different levels according to the hierarchical tree. The root of the graph is defined as level 0 and the outer nodes without any child nodes connected as leaves. The child of a GO term describes more specific biological meanings than the parent. The LC values of leaves without children are defined as 1, and The LC values of the other terms gradually decrease in the proportion of their levels to the levels of their children, as defined in the following formula. Usually, a term (term_*i*_) is associated with several children; thus, all the children would contribute their weights to term_*i*_. The LC value of term_*i*_ is defined as

(8) $$\begin{array}{c} {\text{LC}}_{i}=\frac{1}{n}\overset{n}{\underset{j=1}{\sum }}\left({\text{LC}}_{c_{j}}\frac{\text{Level}\left({\text{term}}_{i}\right)}{\text{Level}\left(c_{j}\right)}\right), \end{array}$$

where *n* denotes the number of the children of term_*i*_ and *c*_*j*_ denotes the children of term_*i*_. It is challenging to calculate the LC of all terms, because leaves can exist in different levels and the levels of the children of a term can also be different (Supplementary Figure 1). We developed a recursion method from leaves to root in order to calculate the LC values of all terms. The LC of a leaf is assigned the value of 1 and written to data set LCs—the recursion begins from the penultimate level. In order to calculate the LC of term_*i*_ in level l, the children of term_*i*_ should have been acquired from the GO database and the LC values of all these children calculated. According to the formula, the LC values of terms in level l can be computed and then written to the data set LCs. The loop repeats until the LC values of terms in all levels are calculated.

### 3.3. LC-Weighted Model to Measure Functional Similarity

The annotation information of a gene product in the GO database contains not only the semantic information of GO terms but also all the ancestors of these annotated terms. If there are *n* GO terms in the annotation database, a vector with length *n* and containing 1 or 0 is defined as the annotation class for a gene, 1 for annotated terms and 0 for unannotated terms. Therefore, the function relationship of two gene products can be measured by analyzing the overlap information of their annotation classes. Because GO terms contain different levels of information, the LC is used to adjust the GO annotation weights of genes to build an agreement table. For two genes, the adjusted agreement table can be built as follows:

(9) t1t2⋮tn(1001⋮⋮00)×(lc1lc2⋮lcn)→sum(p11p21p12p22).

The matrix with 1 and 0 denotes two columns of annotation classes of the two genes and each term (*t*_1_ ⋯ *t*_*n*_) is weighted by its LC (lc_1_ ⋯ lc_*n*_). The *p*_11_ and *p*_22_ in the agreement table denote the adjusted weights for both annotated and unannotated terms, while *p*_12_ and *p*_21_ denote the weights of annotated terms in either gene1 or gene2.

As the contingency table has been weighted by the LC method, the kappa method (instead of weighted-kappa method) can be directly used to estimate the semantic similarity. Specifically, once the adjusted agreement table is obtained, we can use Cohen's kappa coefficient as a statistical measure of interrater agreement for the items in the agreement table. Two items are concordant if the kappa value is greater than 0 and the items are more coincident if the kappa value is closer to 1. The magnitude of terms is large enough to calculate the standard errors of kappa values according to the algorithm by Fleiss et al. [23]. The *Z* test is used to test the significance of kappa values. Two gene products are more significantly similar in function if the *Z* scores are bigger. Consider

(10) $$\begin{array}{c} Z=\frac{\kappa }{SE\left(\kappa \right)}, \end{array}$$

where the kappa and standard deviation of kappa were implemented by the *R* package “vcd” [24]. The *Z* score is used in our method to quantify the similarity between two lists of terms.

### 3.4. Performance Evaluation

The similarity score between a gene and a pathway was calculated by the annotated GO terms of the gene and the overrepresented GO terms of the pathway using the 6 methods evaluated here. For a given pathway, the genes within the pathway should be functionally similar to the enriched GO terms of the pathway. Therefore, the scores between a given pathway and the genes within that pathway, as well as the scores between the pathway and all other genes, were separately calculated—the results were then evaluated using receiver operating characteristics (ROC) curves.

Plotting ROC curves, generally displayed as the true positive rate (TPR) versus the false positive rate (FPR), is a popular means of comparative analysis of computational models and diagnostic biomarkers. For a continuous-scaled marker, the ROC curve graphically depicts the method's discriminatory ability for all threshold values in a unit square by plotting the proportion of true positives (sensitivity) versus the proportion of false positives (1−specificity). The area under the ROC curve (AUC) is the most popular overall discrimination accuracy index and it has been extensively used by many researchers for method evaluation and selection. Typically reasonable classifier systems gain an AUC value of more than 0.5. Greater AUC value indicates greater discriminatory ability of a classifier over all threshold values. The *R* package “ROCR” [25] was used to plot ROC curves and compute the AUC values of these functional similarity methods. Given a pathway, the functional similarity scores between the enriched terms of this pathway and all annotated genes are calculated with different methods. The genes in the pathway are considered as truly positively related, and the genes that do not belong to the pathway are considered as truly negatively related. In this way, the ROC curves help to evaluate the performances of different methods.

## 4. Results

### 4.1. Compilation of the Evaluation Data Set

For a given pathway from the KEGG database, the overrepresented GO terms were identified with functions from the *R* package “topGO” [26]. The top 20 enriched terms in each of the three GO categories were combined to build a group of representative functions for the pathway. The obtained functions for each pathway were used to evaluate the performance of the proposed FSim method and other existing methods. The evaluation results are essentially the same using the top 30 enriched terms from topGO package (data not shown).

### 4.2. The Performance of FSim

**Figure 1:**
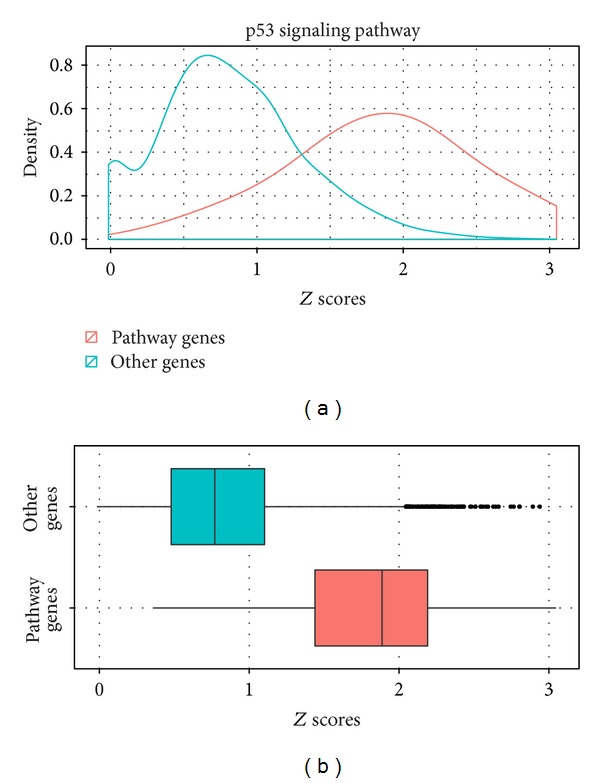
The distribution of FSim-calculated functional similarity scores for comparing the genes in the P53 signaling pathway (in red) with all the other genes (in green). The density plot and box-whisker plots of the two groups of functional similarity scores are shown in (a) and (b), separately. The figures show that the average functional similarity scores for genes within P53 pathway and the other genes which are not in P53 pathway are around 2 and 1, respectively.

**Figure 2:**
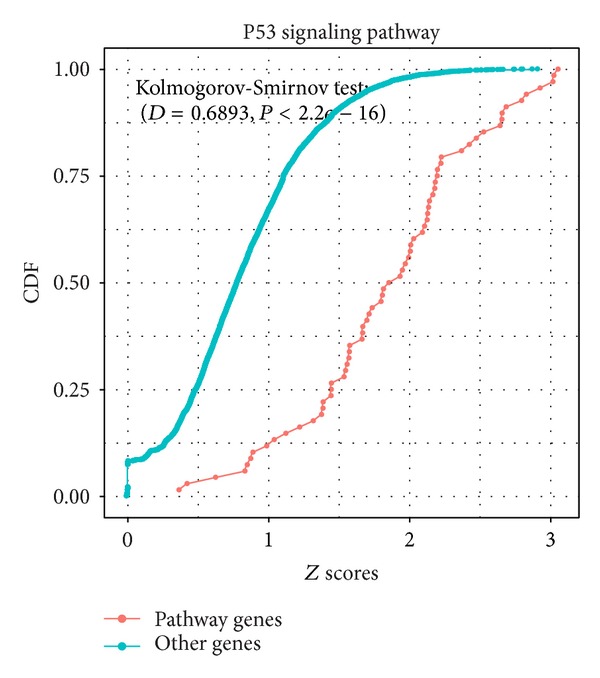
The cumulative distribution functions of the two groups of FSim-calculated functional similarity scores for the P53 pathway. The *x*-axis is the FSim functional similarity scores and the *y*-axis is the cumulative distribution functions. The distribution of the functional similarity scores of the P53 pathway genes (in red) is systematically higher than the distribution of the functional similarity scores of the other genes (in green), with the *P* value of Kolmogorov-Smirnov test less than 2.2*e* − 16.

We first calculated the similarity scores between the group of overrepresented GO terms and all annotated genes from the KEGG database using our proposed FSim method. Given a pathway, the similarity scores for the genes that belong to the pathway and the genes that do not belong to the pathway are computed using FSim method. The rationale is the distribution of the former scores should be higher than the latter scores. The comparison is performed using the* Kolmogorov-Smirnov* (KS) test. Indeed, we found (for all 223 pathways from the KEGG database) that the FSim scores for the genes in the pathway are significantly higher than those for the other genes (Supplementary Table S1). While we obtained similar results on different pathways of KEGG database, we used the p53 signaling pathway as an example to illustrate the performance of FSim. The distribution of similarity scores for the p53 pathway genes and other genes is shown with a density plot in Figure 1(a) and box-whisker plot in Figure 1(b), respectively. The cumulative distributions of the scores are shown in Figure 2. From these two figures, we can see that the distribution of the FSim scores for the genes within P53 pathway is systematically higher than the distribution of the scores of the other genes which are not in the P53 pathway.

**Figure 3:**
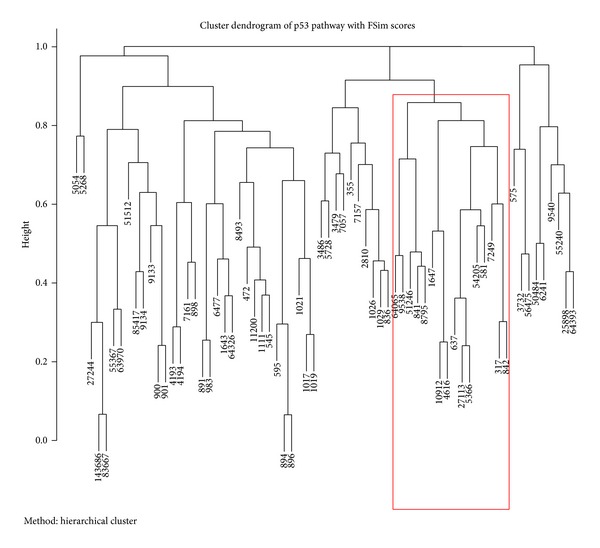
Hierarchical clustering analysis of the p53 signaling pathway genes with FSim functional similarity scores. The Entrez gene ID is shown for each gene in the p53 pathway. The pathway genes are clustered based on the functional similarity distances between each two genes, as calculated by FSim. A subcluster of 13 genes were highlighted in red rectangle. Eleven of these 13 genes were in the same apoptosis pathway except for TSC2 (Entrez ID 7249) and GADD45A (Entrez ID 1647).

The FSim-calculated similarity scores can be used to classify adjacent genes in a pathway into functional subgroups. The p53 pathway from KEGG database was used again as an example. The similarity score for each pair of the 68 genes in the pathway was calculated using the FSim method. The obtained similarity score between genes was clustered using hierarchical clustering algorithm and shown in Figure 3. From the plot we can see that there were 13 genes in a highlighted cluster. Eleven of these 13 genes were in the same apoptosis pathway except for TSC2 and GADD45A. Three pairs of adjacent gene products in the pathway were classified to the smallest subgroups, including DR5(8795)/CASP8(841), Noxa(5366)/PUMA(27113), and Apaf-1(317)/CASP9(842), which are consistent with the manual curation provided by KEGG database.

### 4.3. The Comparison between FSim and Other Methods

**Figure 4:**
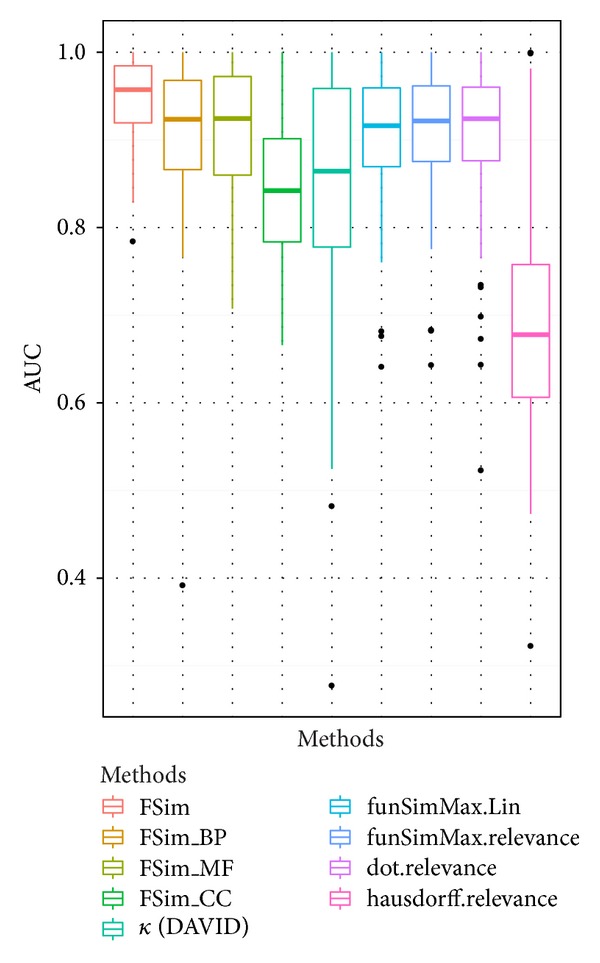
Performance comparison of different algorithms using all pathway data sets. The box-whisker plots show the AUC value distributions of the FSim method using separate BP (FSim_BP) ontology, MF (FSim_MF) ontology, and CC (FSim_CC) ontology and using all three ontologies (FSim), as well as the other five evaluated methods. The band in each box is the median AUC values of each method, which can be used to compare the overall performance of all the methods.

In order to compare the performance of FSim with other methods, we also calculated the similarity scores between the group of overrepresented GO terms and all annotated genes from the KEGG database using other methods. The kappa statistical method used in the DAVID classification tool [18] measures the functional agreement of GO annotation. The four IC based methods used in the comparison include funSimMax with Lin's method [11] and relevance methods [19] separately, the dot product between feature vectors (dot) [20], and hausdorff distance [15]. The sum of similarity scores from the 3 ontologies is used for the IC based methods. The ROC curve for a given pathway was generated where the genes within the pathway are considered as true positive and the genes that do not belong to the pathway are considered as true negative. The AUC is used to compare the performance between different methods. As summarized in Supplementary Table S1, the AUC of FSim is better than all the other methods in 164 out of 229 pathways. The box-whisker plots in Figure 4 show the AUC values of the FSim method using separate BP (FSim_BP) ontology, separate MF (FSim_MF) ontology, and separate CC (FSim_CC) ontology and using all three ontologies (FSim)—the other 5 compared methods are also plotted. All methods were capable of identifying genes that are functionally similar as all the AUC values were more than 0.5. The FSim method using all three ontologies, however, has an overall better performance than the other methods.

**Figure 5:**
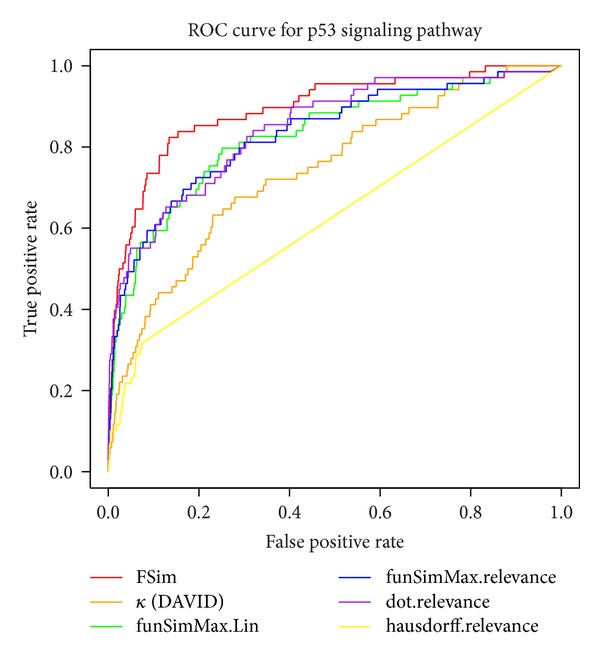
The ROC curve for the performance comparison of different algorithms using the p53 signaling pathway as an exemplary evaluation data set. The AUC value for FSim in the P53 pathway is 0.89, compared with 0.74, 0.83, 0.83, 0.85, and 0.62 for DAVID, funSimMax. Lin, funSimMax.relevance, dot.relevance, and hausdorff.relevance, respectively.

For the purpose of visualizing the results of performance comparisons, the results for the P53 signaling pathway were used to generate ROC curves for the FSim algorithm and the other five functional similarity methods (Figure 5). The AUC value for FSim in the P53 pathway is 0.89, and the second best value is 0.85 for the “dot.relevence” method.

## 5. Implementation

**Figure 6:**
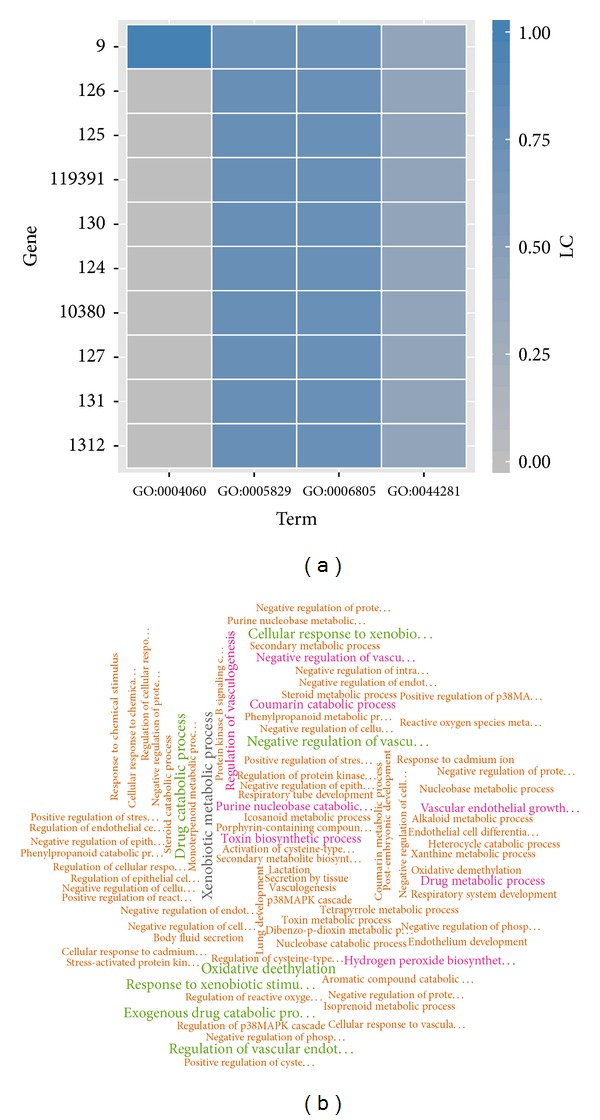
The two exemplary visualization plots provided by FSim package. The heat map in (a) displays whether the genes are annotated to a given group of GO terms. A total of 10 genes and 4 GO terms are displayed in this heat map. The *y*-axis is the Entrez gene ID, and the *x*-axis is the GO term ID. The squares in the map denote whether a gene is annotated with a corresponding GO term, with the color proportional to the LC scores. The word cloud in (b) displays all overrepresented GO terms for a given pathway. The GO terms are listed with different text scales and colors corresponding to their respective enriched weights.

The FSim package has been implemented as a package within the statistical environment *R* and is distributed under GPL license under the *R*-forge project [27]. The data packages, such as “GO.db” and “org.Hs.eg.db,” are required as the source of gene annotations. The Entrez gene IDs or official symbols are required for retrieving GO annotations. The functions for GO overrepresented analysis from “topGO” package are integrated into the FSim package for gene set studies. A core function “SearchGene” was developed to search functionally related genes for a gene, a group of GO terms, or even a list of biological functions. A number of functions are available to visualize the results. For example, heat map can be used to display the shared GO terms of the top functional related genes (Figure 6(a)). The overrepresented GO terms of a gene set can be also visualized using word cloud (Figure 6(b)). More options, such as number of terms to be plotted, can be specified to optimize the display of the top enriched terms. A table of enriched GO terms with annotation and scores for a given pathway can also be generated by existing functions in the FSim package. In addition, customized GO annotations, as well as annotation data sets prepared by users or from other databases, can be directly used in our package.

## 6. Discussion

We have developed an algorithm to measure the functional similarity of gene products based on multiple ontologies of GO annotation. Our LC-weighted model not only integrates the information of the specific GO terms, but also considers the hierarchy of structures among them. We have shown that the similarity scores calculated based on our LC-weighted methods are capable of determining whether genes are related with certain biological functions. The evaluation, using the validation set derived from the curated KEGG pathway database, showed that our method performs better than several representative methods available to the public. The novelty of our method lies in the use of LC to adjust the GO annotation weights of genes. Traditionally, IC based algorithms have been used to measure the functional relationship of gene products [15, 16, 19, 28]. The IC value is a good choice to measure the semantic similarity of GO terms. Several studies have evaluated IC based algorithms [29–31]. Resnik's and Lin's methods with average of the best match (funSimMax) performed better than other methods in most of these evaluations. The Schlicker's method (relevance) combines Lin's and Resnik's similarity measures to take relevant information into account [19]. Four IC based methods, including funSimMax and the relevance method, are selected in our evaluation. The evaluation results showed that the IC based methods have similar performance. The FSim method performed better than the IC based methods, suggesting that our LC based method was a promising alternative for the traditional IC based measures. In addition, the IC values depend on the probability of GO terms in the annotation databases, which changes with the inclusion of different annotation databases and even different version of the same database. For example, when we try to discover the functional relation between a gene product from the GOA database [8] and a protein domain from Pfam [32], two different lists of GO terms will be obtained from the respective databases. These two lists are not comparable in an IC based approach because their probabilities are calculated from different databases. In contrast, the FSim method uses LC values to measure the specificities of GO terms, which only depend on the GO database. Therefore, our LC based methods are more robust and versatile.

Another novelty of our algorithm is that it takes advantage of all GO terms in three ontologies, rather than a single ontology. In our evaluation we have shown that the FSim method using all ontologies performed better than the same method using a single ontology. The methods using BP or MF ontology had similar performances and both of them performed better than the method using CC. In the current version of GO database, BP, MF, and CC ontology contained 25193, 9602, and 3232 terms, respectively. Therefore the BP and MF ontologies provide more specific and explicit biological knowledge to annotate gene products. The existing gene functional similarity methods only adopt the terms from a single ontology; as a result, many gene products are annotated with the same GO terms in a single ontology. For example, according to the current GO annotation database, there are only 10903 distinct GO terms in the BP ontology for a total 46265 gene products in human species. Therefore, using a single ontology to annotate the functional relationship of gene products will likely miss important annotation information provided by other ontologies.

We will continue extending the functionality of FSim method. For example, the calculation of LC used the hierarchical structure of GO terms to define their relationships. In the GO term database, the relations between GO terms are categorized and defined as “is a,” “part of,” and “regulates.” The current implementation does not distinguish their difference. Besides, the annotation for gene products comes with an evidence code to indicate how the annotation was supported. Our current approach allows the user to either choose to use high-confidence annotations or filter out terms with low evidence (e.g., inferred from electronic annotation (IEA)) for their specific application. A future improvement is to provide a weighted approach to the different evidence codes.

## 7. Conclusion

We proposed a novel LC-weighted algorithm to measure the functional similarity of gene products with GO annotations with hierarchical structures. Our method achieves better performance than the other methods in the evaluation. The method is implemented as a package in the statistical computing environment *R* and it is freely available under the *R*-forge project (http://fsim.r-forge.r-project.org/). The package provides flexible functions to search functional related gene products with a given gene product, a gene set, or even a list of biological key words as input. It is a flexible and powerful tool for rapidly discovering functionally similar gene products from fast-expanding GO annotation databases.

## Supplementary Material

The supplementary material includes a directed acyclic graph to demonstrate the hierarchical structure of GO terms and a table that contains the evaluation results of all the KEGG pathway data sets. In the supplementary figure 1, the children-parents relationship of the GO terms and the leaf and root terms are shown. The supplementary table 1 lists the KS test results of FSim method, as well as the AUC values of the FSim and the other compared methods using all the KEGG pathway data sets.

## References

[1] Ashburner M, Ball CA, Blake JA (2000). Gene ontology: tool for the unification of biology. *Nature Genetics*.

[2] Kanehisa M, Goto S (2000). KEGG: kyoto encyclopedia of genes and genomes. *Nucleic Acids Research*.

[3] Croft D, O'Kelly G, Wu G (2011). Reactome: a database of reactions, pathways and biological processes. *Nucleic Acids Research*.

[4] Boutet E, Lieberherr D, Tognolli M, Schneider M, Bairoch A (2007). UniProtKB/Swiss-Prot: the manually annotated section of the UniProt KnowledgeBase. *Methods in Molecular Biology*.

[5] UniProt Consortium (2013). Update on activities at the Universal Protein Resource (UniProt) in 2013. *Nucleic Acids Research*.

[6] Letunic I, Doerks T, Bork P (2009). SMART 6: recent updates and new developments. *Nucleic Acids Research*.

[7] Amberger J, Bocchini C, Hamosh A (2011). A new face and new challenges for Online Mendelian Inheritance in Man (OMIM^®^). *Human Mutation*.

[8] Barrell D, Dimmer E, Huntley RP, Binns D, O'Donovan C, Apweiler R (2009). The GOA database in 2009—an integrated Gene Ontology Annotation resource. *Nucleic Acids Research*.

[9] Dimmer EC, Huntley RP, Alam-Faruque Y (2012). The UniProt-GO Annotation database in 2011. *Nucleic Acids Research*.

[10] Resnik P (1999). Semantic similarity in a taxonomy: an information-based measure and its application to problems of ambiguity in natural language. *Journal of Artificial Intelligence Research*.

[11] Lin D An information-theoretic definition of similarity.

[12] Jiang JJ, Conrath DW Semantic similarity based on corpus statistics and lexical taxonomy.

[13] Schlicker A, Rahnenführer J, Albrecht M, Lengauer T, Domingues FS (2007). GOTax: investigating biological processes and biochemical activities along the taxonomic tree. *Genome Biology*.

[14] Du Z, Li L, Chen C, Yu PS, Wang JZ (2009). G-SESAME: web tools for GO-term-based gene similarity analysis and knowledge discovery. *Nucleic Acids Research*.

[15] Fröhlich H, Speer N, Poustka A, Beißbarth T (2007). GOSim—an R-package for computation of information theoretic GO similarities between terms and gene products. *BMC Bioinformatics*.

[16] Schlicker A, Albrecht M (2009). FunSimMat update: new features for exploring functional similarity. *Nucleic Acids Research*.

[17] Schlicker A, Albrecht M (2008). FunSimMat: a comprehensive functional similarity database. *Nucleic Acids Research*.

[18] Huang DW, Sherman BT, Tan Q (2007). The DAVID gene functional classification tool: a novel biological module-centric algorithm to functionally analyze large gene lists. *Genome Biology*.

[19] Schlicker A, Domingues FS, Rahnenführer J, Lengauer T (2006). A new measure for functional similarity of gene products based on gene ontology. *BMC Bioinformatics*.

[20] Mistry M, Pavlidis P (2008). Gene ontology term overlap as a measure of gene functional similarity. *BMC Bioinformatics*.

[21] del Pozo A, Pazos F, Valencia A (2008). Defining functional distances over gene ontology. *BMC Bioinformatics*.

[22] Gentleman RC, Carey VJ, Bates DM (2004). Bioconductor: open software development for computational biology and bioinformatics. *Genome Biology*.

[23] Fleiss JL, Cohen J, Everitt BS (1969). Large sample standard errors of kappa and weighted kappa. *Psychological Bulletin*.

[24] Meyer D, Zeileis A, Hornik K (2006). The strucplot framework: visualizing multi-way contingency tables with vcd. *Journal of Statistical Software*.

[25] Sing T, Sander O, Beerenwinkel N, Lengauer T (2005). ROCR: visualizing classifier performance in R. *Bioinformatics*.

[26] Alexa A, Rahnenführer J, Lengauer T (2006). Improved scoring of functional groups from gene expression data by decorrelating GO graph structure. *Bioinformatics*.

[27] Theußl S, Zeileis A (2009). Collaborative software development using R-forge. *The R Journal*.

[28] Zhang P, Zhang J, Sheng H, Russo JJ, Osborne B, Buetow K (2006). Gene functional similarity search tool (GFSST). *BMC Bioinformatics*.

[29] Guo X, Liu R, Shriver CD, Hu H, Liebman MN (2006). Assessing semantic similarity measures for the characterization of human regulatory pathways. *Bioinformatics*.

[30] Xu T, Du LF, Zhou Y (2008). Evaluation of GO-based functional similarity measures using *S. cerevisiae* protein interaction and expression profile data. *BMC Bioinformatics*.

[31] Pesquita C, Faria D, Bastos H, Ferreira AEN, Falcão AO, Couto FM (2008). Metrics for GO based protein semantic similarity: a systematic evaluation. *BMC Bioinformatics*.

[32] Punta M, Coggill PC, Eberhardt RY (2012). The Pfam protein families database. *Nucleic Acids Research*.

