# Supplementary material for: FSim: A Novel Functional Similarity Search Algorithm and Tool for Discovering Functionally Related Gene Products
Source: Biomed Res Int. 2014 Aug 12;2014:509149. doi: 10.1155/2014/509149 (PMC4145548; doi:10.1155/2014/509149)
Supplement: Supplementary file 1 — The supplementary material includes a directed acyclic graph to demonstrate the hierarchical structure of GO terms and a table that contains the evaluation results of all the KEGG pathway data sets. In the supplementary figure 1, the children-parents relationship of the GO terms and the leaf and root terms are shown. The supplementary table 1 lists the KS test results of FSim method, as well as the AUC values of the FSim and the other compared methods using all the KEGG pathway data sets. [file 509149.f1.pdf]

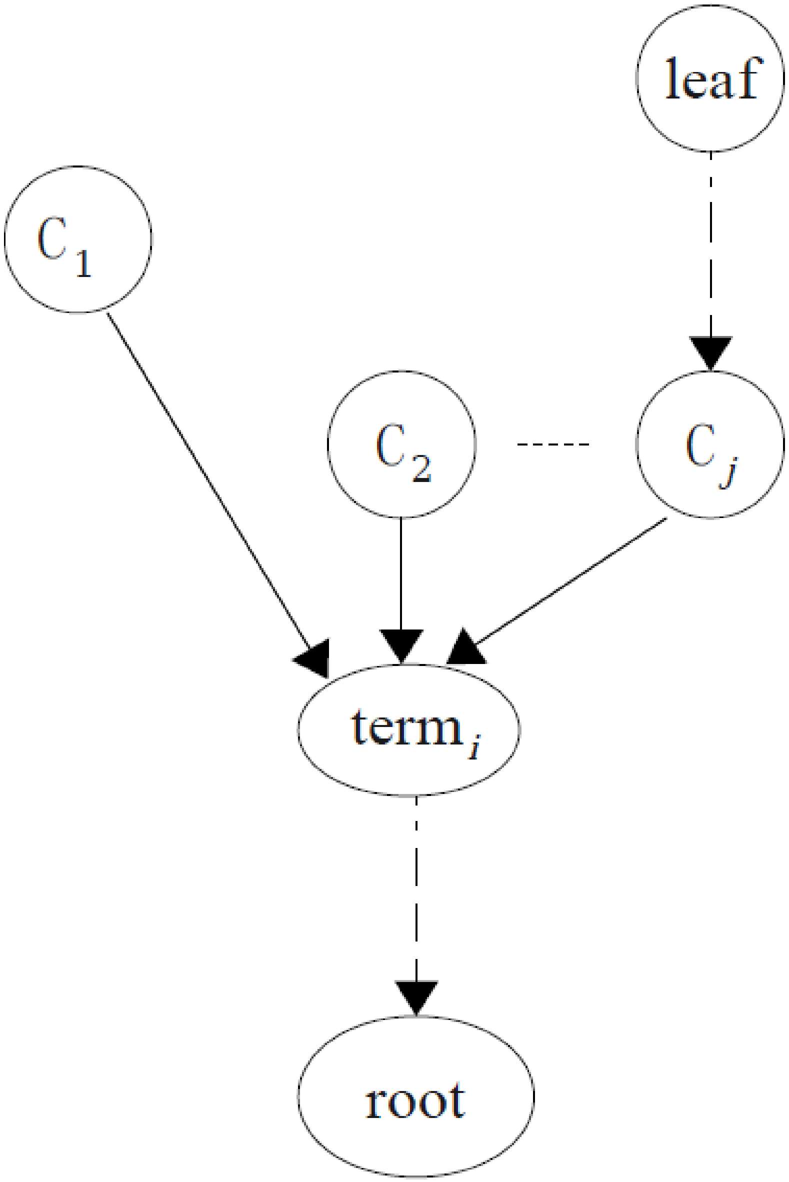

Supplementary Figure 1. The relationship of the GO terms. C_j denotes one of the children of termi. C_1 is in different levels with C_2. Root denotes the node without parents and leaf denotes the node without children.

Supplementary Table 1. KS test results of FSim and AUC values of FSim and the compared methods.

KEGG pathway	FSim KS Test P Value	FSim KS Test Statistic Value	AUC Fsim BP	Fsim MF	Fsim CC	Fsim ALL	Kappa (DAVID)	funSim MaxLin	funSimMax relevance	dot relevance	hausdorff relevance
hsa00232	3.56E-06	0.973	0.935	0.991	0.869	0.990	0.988	0.994	0.996	0.984	0.758
hsa00983	0.00E+00	0.775	0.937	0.914	0.787	0.952	0.923	0.931	0.927	0.945	0.635
hsa01100	0.00E+00	0.663	0.875	0.842	0.799	0.894	0.833	0.869	0.885	0.818	0.589
hsa00230	0.00E+00	0.748	0.884	0.940	0.692	0.942	0.872	0.889	0.902	0.931	0.733
hsa05340	1.81E-14	0.682	0.936	0.803	0.756	0.898	0.814	0.835	0.836	0.837	0.655
hsa04514	0.00E+00	0.811	0.927	0.822	0.927	0.965	0.854	0.907	0.917	0.861	0.617
hsa05412	0.00E+00	0.842	0.950	0.928	0.901	0.974	0.915	0.953	0.955	0.964	0.607
hsa04010	0.00E+00	0.588	0.788	0.708	0.738	0.863	0.696	0.824	0.823	0.852	0.631
hsa04012	0.00E+00	0.681	0.817	0.869	0.828	0.901	0.661	0.878	0.891	0.889	0.618
hsa04062	0.00E+00	0.712	0.859	0.862	0.750	0.926	0.671	0.866	0.861	0.894	0.578
hsa04150	0.00E+00	0.848	0.902	0.879	0.904	0.962	0.786	0.923	0.931	0.960	0.533
hsa04210	0.00E+00	0.665	0.843	0.861	0.767	0.895	0.729	0.854	0.866	0.898	0.631
hsa04370	0.00E+00	0.793	0.892	0.871	0.800	0.929	0.777	0.858	0.873	0.893	0.589
hsa04380	0.00E+00	0.588	0.812	0.809	0.735	0.851	0.656	0.814	0.822	0.856	0.631
hsa04510	0.00E+00	0.669	0.888	0.807	0.797	0.901	0.748	0.836	0.838	0.889	0.563
hsa04530	0.00E+00	0.785	0.838	0.837	0.896	0.936	0.883	0.915	0.913	0.903	0.623
hsa04620	0.00E+00	0.839	0.957	0.873	0.775	0.973	0.744	0.939	0.941	0.969	0.673
hsa04630	0.00E+00	0.671	0.848	0.888	0.746	0.905	0.645	0.882	0.883	0.892	0.555
hsa04660	0.00E+00	0.681	0.874	0.815	0.837	0.905	0.695	0.874	0.883	0.906	0.644
hsa04662	0.00E+00	0.738	0.829	0.819	0.868	0.910	0.713	0.897	0.901	0.862	0.690
hsa04664	0.00E+00	0.803	0.885	0.860	0.798	0.930	0.724	0.924	0.934	0.945	0.604
hsa04666	0.00E+00	0.677	0.842	0.876	0.840	0.913	0.824	0.904	0.907	0.857	0.622
hsa04722	0.00E+00	0.629	0.825	0.796	0.845	0.877	0.603	0.843	0.852	0.860	0.570
hsa04910	0.00E+00	0.732	0.909	0.813	0.838	0.909	0.808	0.857	0.867	0.891	0.631
hsa04914	0.00E+00	0.770	0.886	0.840	0.820	0.947	0.804	0.905	0.910	0.947	0.606
hsa04920	0.00E+00	0.618	0.834	0.847	0.783	0.852	0.698	0.850	0.856	0.876	0.608
hsa04973	0.00E+00	0.840	0.895	0.899	0.818	0.967	0.803	0.884	0.893	0.842	0.685
hsa05142	0.00E+00	0.656	0.809	0.875	0.793	0.898	0.587	0.852	0.852	0.943	0.589
hsa05145	0.00E+00	0.664	0.807	0.796	0.751	0.906	0.659	0.802	0.805	0.891	0.560
hsa05160	0.00E+00	0.749	0.852	0.818	0.768	0.941	0.795	0.843	0.867	0.923	0.675
hsa05200	0.00E+00	0.563	0.767	0.841	0.745	0.838	0.483	0.806	0.817	0.868	0.570
hsa05210	0.00E+00	0.709	0.766	0.880	0.867	0.911	0.525	0.880	0.897	0.942	0.636
hsa05211	0.00E+00	0.751	0.875	0.872	0.830	0.936	0.751	0.899	0.903	0.893	0.605
hsa05212	0.00E+00	0.682	0.818	0.901	0.837	0.912	0.589	0.862	0.878	0.929	0.531
hsa05213	0.00E+00	0.775	0.810	0.925	0.869	0.938	0.583	0.900	0.916	0.955	0.572
hsa05214	0.00E+00	0.607	0.829	0.828	0.835	0.876	0.626	0.816	0.824	0.845	0.654

hsa05215	0.00E+00	0.662	0.829	0.890	0.791	0.881	0.569	0.846	0.856	0.927	0.566
hsa05218	0.00E+00	0.816	0.896	0.912	0.772	0.961	0.687	0.923	0.929	0.944	0.704
hsa05220	0.00E+00	0.661	0.807	0.889	0.830	0.892	0.526	0.891	0.902	0.914	0.577
hsa05221	0.00E+00	0.742	0.866	0.904	0.842	0.920	0.632	0.882	0.889	0.922	0.625
hsa05222	0.00E+00	0.762	0.822	0.898	0.909	0.950	0.657	0.867	0.872	0.941	0.514
hsa05223	0.00E+00	0.760	0.851	0.909	0.885	0.938	0.703	0.920	0.928	0.933	0.559
hsa04146	0.00E+00	0.846	0.916	0.893	0.964	0.978	0.938	0.952	0.961	0.965	0.639
hsa00520	0.00E+00	0.806	0.931	0.925	0.760	0.953	0.807	0.934	0.944	0.914	0.765
hsa04974	0.00E+00	0.805	0.949	0.964	0.843	0.963	0.778	0.969	0.971	0.843	0.502
hsa04622	0.00E+00	0.830	0.953	0.823	0.730	0.964	0.802	0.920	0.921	0.914	0.787
hsa03013	0.00E+00	0.826	0.943	0.873	0.894	0.964	0.892	0.886	0.879	0.858	0.786
hsa04145	0.00E+00	0.718	0.904	0.834	0.830	0.920	0.830	0.828	0.839	0.833	0.664
hsa04612	0.00E+00	0.604	0.866	0.776	0.784	0.838	0.757	0.775	0.783	0.765	0.711
hsa04672	0.00E+00	0.813	0.939	0.856	0.850	0.963	0.829	0.904	0.912	0.910	0.623
hsa04940	0.00E+00	0.830	0.888	0.884	0.880	0.957	0.811	0.863	0.874	0.918	0.709
hsa05140	0.00E+00	0.745	0.907	0.870	0.750	0.936	0.728	0.869	0.871	0.920	0.747
hsa05150	0.00E+00	0.813	0.929	0.858	0.830	0.956	0.874	0.912	0.918	0.902	0.656
hsa05310	2.22E-16	0.783	0.944	0.906	0.849	0.932	0.778	0.909	0.918	0.913	0.671
hsa05320	0.00E+00	0.808	0.935	0.917	0.834	0.958	0.860	0.909	0.911	0.896	0.683
hsa05322	0.00E+00	0.793	0.937	0.858	0.851	0.957	0.858	0.677	0.684	0.699	0.535
hsa05323	0.00E+00	0.744	0.877	0.855	0.938	0.945	0.670	0.913	0.918	0.906	0.529
hsa05330	0.00E+00	0.838	0.966	0.904	0.868	0.972	0.854	0.891	0.901	0.924	0.839
hsa05332	0.00E+00	0.778	0.948	0.902	0.897	0.953	0.800	0.889	0.897	0.870	0.756
hsa05416	0.00E+00	0.732	0.828	0.830	0.878	0.933	0.861	0.817	0.827	0.906	0.710
hsa04640	0.00E+00	0.826	0.892	0.859	0.904	0.956	0.831	0.921	0.924	0.866	0.651
hsa00564	0.00E+00	0.800	0.955	0.978	0.668	0.954	0.899	0.924	0.936	0.947	0.692
hsa00565	0.00E+00	0.914	0.979	0.980	0.719	0.987	0.864	0.962	0.964	0.933	0.491
hsa00590	0.00E+00	0.839	0.932	0.970	0.793	0.971	0.860	0.958	0.958	0.925	0.508
hsa00591	0.00E+00	0.926	0.992	0.991	0.764	0.984	0.966	0.955	0.958	0.945	0.739
hsa00592	1.11E-16	0.991	0.994	0.996	0.717	0.999	0.959	0.948	0.949	0.930	0.767
hsa04270	0.00E+00	0.697	0.852	0.834	0.755	0.924	0.756	0.816	0.818	0.807	0.663
hsa04730	0.00E+00	0.748	0.870	0.870	0.733	0.928	0.816	0.834	0.851	0.881	0.722
hsa04912	0.00E+00	0.636	0.824	0.815	0.713	0.852	0.762	0.801	0.819	0.852	0.656
hsa04972	0.00E+00	0.729	0.839	0.887	0.760	0.940	0.685	0.916	0.919	0.808	0.656
hsa04975	0.00E+00	0.811	0.979	0.943	0.701	0.969	0.857	0.948	0.951	0.932	0.536
hsa04144	0.00E+00	0.679	0.844	0.765	0.780	0.916	0.780	0.803	0.799	0.861	0.703
hsa04310	0.00E+00	0.651	0.836	0.816	0.771	0.898	0.667	0.819	0.823	0.863	0.667
hsa00563	0.00E+00	0.959	0.999	0.935	0.967	0.995	0.974	0.996	0.996	1.000	0.940
hsa03008	0.00E+00	0.715	0.812	0.730	0.901	0.916	0.898	0.804	0.820	0.800	0.748
hsa04141	0.00E+00	0.745	0.883	0.763	0.796	0.938	0.846	0.819	0.825	0.814	0.709
hsa03420	0.00E+00	0.912	0.990	0.950	0.964	0.993	0.989	0.960	0.963	0.968	0.890
hsa04120	0.00E+00	0.795	0.894	0.929	0.757	0.955	0.888	0.886	0.903	0.898	0.628
hsa04670	0.00E+00	0.743	0.912	0.879	0.874	0.942	0.812	0.899	0.908	0.913	0.599

hsa03410	0.00E+00	0.955	0.989	0.996	0.924	0.996	0.979	0.959	0.960	0.953	0.749
hsa03010	0.00E+00	0.983	0.990	0.984	0.998	0.998	0.992	0.962	0.964	0.954	0.952
hsa04110	0.00E+00	0.786	0.946	0.826	0.904	0.953	0.861	0.916	0.924	0.928	0.802
hsa04114	0.00E+00	0.663	0.846	0.815	0.796	0.895	0.792	0.809	0.811	0.841	0.617
hsa04350	0.00E+00	0.710	0.916	0.883	0.791	0.930	0.741	0.872	0.871	0.867	0.668
hsa04710	2.00E-15	0.888	0.970	0.845	0.893	0.984	0.982	0.933	0.935	0.938	0.848
hsa05130	0.00E+00	0.709	0.854	0.868	0.896	0.913	0.818	0.862	0.865	0.862	0.712
hsa00140	0.00E+00	0.882	0.988	0.968	0.878	0.981	0.980	0.951	0.951	0.959	0.606
hsa04621	0.00E+00	0.738	0.934	0.851	0.765	0.936	0.758	0.890	0.895	0.899	0.640
hsa04142	0.00E+00	0.871	0.816	0.884	0.953	0.970	0.869	0.910	0.924	0.838	0.632
hsa00970	0.00E+00	0.965	0.983	0.981	0.904	0.990	0.988	0.642	0.643	0.644	0.677
hsa02010	0.00E+00	0.993	0.968	0.998	0.868	0.999	0.906	0.986	0.989	0.887	0.542
hsa05100	0.00E+00	0.798	0.863	0.926	0.879	0.950	0.814	0.913	0.914	0.878	0.602
hsa03320	0.00E+00	0.687	0.869	0.910	0.755	0.903	0.823	0.884	0.886	0.905	0.491
hsa00190	0.00E+00	0.964	0.973	0.974	0.963	0.993	0.996	0.877	0.878	0.875	0.801
hsa03040	0.00E+00	0.899	0.956	0.809	0.949	0.981	0.964	0.943	0.946	0.912	0.723
hsa00532	0.00E+00	0.979	0.927	0.978	0.986	0.997	0.993	0.993	0.994	0.995	0.855
hsa04810	0.00E+00	0.671	0.866	0.841	0.757	0.909	0.758	0.854	0.868	0.876	0.551
hsa05131	0.00E+00	0.713	0.896	0.870	0.851	0.913	0.818	0.889	0.890	0.885	0.613
hsa03440	0.00E+00	0.979	0.995	0.983	0.917	0.995	0.984	0.985	0.986	0.985	0.892
hsa03450	4.35E-11	0.973	0.995	0.988	0.900	0.996	0.994	0.922	0.923	0.923	0.819
hsa05016	0.00E+00	0.605	0.831	0.835	0.853	0.889	0.836	0.794	0.794	0.810	0.695
hsa00760	4.32E-14	0.811	0.913	0.954	0.797	0.961	0.889	0.955	0.949	0.928	0.778
hsa03018	0.00E+00	0.673	0.850	0.860	0.858	0.898	0.862	0.864	0.858	0.872	0.783
hsa04360	0.00E+00	0.734	0.872	0.727	0.712	0.912	0.865	0.878	0.880	0.876	0.648
hsa00300	4.97E-03	1.000	1.000	0.995	0.995	1.000	1.000	0.999	0.998	1.000	0.841
hsa00310	0.00E+00	0.908	0.927	0.978	0.921	0.989	0.938	0.958	0.958	0.950	0.751
hsa04080	0.00E+00	0.806	0.904	0.940	0.903	0.952	0.913	0.914	0.918	0.909	0.663
hsa04520	0.00E+00	0.773	0.897	0.902	0.873	0.947	0.780	0.877	0.888	0.938	0.609
hsa00533	7.58E-13	0.978	0.982	0.998	0.993	0.993	0.991	0.996	0.996	0.994	0.600
hsa04115	0.00E+00	0.689	0.896	0.778	0.793	0.894	0.743	0.827	0.832	0.847	0.623
hsa00830	0.00E+00	0.848	0.963	0.958	0.822	0.955	0.958	0.937	0.937	0.953	0.655
hsa03015	0.00E+00	0.705	0.819	0.897	0.823	0.920	0.839	0.880	0.875	0.841	0.669
hsa05219	0.00E+00	0.779	0.878	0.910	0.822	0.941	0.666	0.896	0.906	0.943	0.519
hsa00510	0.00E+00	0.981	0.995	0.969	0.931	0.998	0.994	0.992	0.993	0.999	0.889
hsa03050	0.00E+00	0.954	0.975	0.793	0.969	0.979	0.965	0.942	0.954	0.960	0.948
hsa05010	0.00E+00	0.587	0.838	0.852	0.831	0.882	0.811	0.760	0.776	0.840	0.679
hsa05120	0.00E+00	0.608	0.849	0.861	0.779	0.870	0.722	0.842	0.838	0.870	0.678
hsa00240	0.00E+00	0.762	0.941	0.924	0.778	0.955	0.916	0.913	0.922	0.919	0.828
hsa04130	0.00E+00	0.895	0.977	0.881	0.911	0.982	0.967	0.963	0.971	0.940	0.723
hsa00130	1.72E-06	0.999	1.000	0.985	0.931	1.000	0.997	0.995	0.994	0.999	0.708
hsa04976	0.00E+00	0.703	0.922	0.849	0.814	0.919	0.839	0.913	0.915	0.850	0.577
hsa05217	0.00E+00	0.877	0.971	0.976	0.904	0.984	0.724	0.939	0.947	0.988	0.789

hsa04623	0.00E+00	0.792	0.966	0.914	0.715	0.964	0.865	0.931	0.934	0.929	0.767
hsa04966	0.00E+00	0.913	0.991	0.977	0.915	0.993	0.993	0.993	0.994	0.992	0.904
hsa05110	0.00E+00	0.679	0.869	0.817	0.846	0.901	0.887	0.880	0.882	0.905	0.741
hsa00601	0.00E+00	0.989	0.992	0.997	0.993	0.997	0.995	0.997	0.997	0.998	0.916
hsa00603	5.27E-12	0.977	0.988	0.998	0.961	0.994	0.991	0.995	0.995	0.998	0.840
hsa04512	0.00E+00	0.847	0.921	0.887	0.942	0.972	0.865	0.940	0.950	0.937	0.593
hsa05146	0.00E+00	0.658	0.840	0.848	0.831	0.905	0.640	0.835	0.843	0.904	0.538
hsa00010	0.00E+00	0.902	0.991	0.945	0.830	0.990	0.926	0.958	0.960	0.979	0.709
hsa00561	0.00E+00	0.792	0.898	0.975	0.693	0.953	0.905	0.964	0.960	0.944	0.579
hsa05216	1.85E-12	0.693	0.858	0.852	0.844	0.907	0.802	0.850	0.848	0.857	0.745
hsa04060	0.00E+00	0.747	0.833	0.952	0.861	0.933	0.663	0.943	0.947	0.895	0.518
hsa00380	0.00E+00	0.875	0.923	0.938	0.869	0.970	0.928	0.939	0.943	0.949	0.678
hsa00270	0.00E+00	0.856	0.925	0.935	0.821	0.970	0.886	0.939	0.931	0.964	0.736
hsa00430	4.73E-08	0.938	0.944	0.997	0.825	0.994	0.982	0.960	0.964	0.993	0.749
hsa04260	0.00E+00	0.933	0.949	0.967	0.934	0.988	0.969	0.910	0.912	0.906	0.618
hsa05410	0.00E+00	0.773	0.924	0.895	0.905	0.952	0.868	0.920	0.931	0.945	0.732
hsa05414	0.00E+00	0.841	0.958	0.866	0.888	0.965	0.878	0.923	0.927	0.925	0.694
hsa04540	0.00E+00	0.657	0.932	0.800	0.863	0.913	0.858	0.869	0.878	0.915	0.660
hsa00920	8.85E-11	0.998	0.993	1.000	0.928	1.000	0.977	0.921	0.921	0.923	0.501
hsa04070	0.00E+00	0.826	0.909	0.955	0.746	0.965	0.876	0.902	0.911	0.897	0.695
hsa04720	0.00E+00	0.727	0.863	0.907	0.832	0.926	0.783	0.857	0.858	0.844	0.720
hsa00562	0.00E+00	0.884	0.892	0.972	0.774	0.971	0.848	0.898	0.912	0.941	0.722
hsa00062	2.96E-06	0.917	0.929	0.991	0.928	0.977	0.938	0.994	0.994	0.974	0.787
hsa00071	0.00E+00	0.918	0.971	0.972	0.922	0.985	0.976	0.975	0.975	0.983	0.693
hsa00280	0.00E+00	0.878	0.954	0.976	0.937	0.985	0.969	0.977	0.977	0.972	0.772
hsa04650	0.00E+00	0.629	0.854	0.776	0.735	0.886	0.716	0.843	0.854	0.812	0.626
hsa05014	0.00E+00	0.748	0.822	0.862	0.842	0.918	0.815	0.850	0.864	0.881	0.527
hsa05012	0.00E+00	0.758	0.874	0.896	0.909	0.916	0.891	0.787	0.787	0.791	0.730
hsa04916	0.00E+00	0.619	0.805	0.836	0.759	0.882	0.651	0.819	0.820	0.884	0.676
hsa04962	0.00E+00	0.782	0.850	0.834	0.793	0.952	0.835	0.897	0.910	0.842	0.635
hsa04140	0.00E+00	0.895	0.954	0.944	0.860	0.988	0.900	0.953	0.952	0.944	0.546
hsa03030	0.00E+00	0.920	0.982	0.943	0.949	0.974	0.990	0.971	0.975	0.963	0.882
hsa00600	0.00E+00	0.910	0.992	0.924	0.818	0.985	0.945	0.986	0.987	0.979	0.672
hsa00100	9.88E-14	0.899	0.995	0.959	0.891	0.984	0.949	0.984	0.986	0.964	0.740
hsa04977	2.49E-14	0.818	0.929	0.804	0.802	0.962	0.830	0.908	0.912	0.862	0.519
hsa00514	0.00E+00	0.887	0.922	0.969	0.886	0.978	0.959	0.946	0.956	0.940	0.701
hsa00450	3.90E-09	0.769	0.947	0.991	0.707	0.923	0.880	0.966	0.972	0.964	0.473
hsa00670	1.05E-13	0.950	0.968	0.995	0.915	0.992	0.963	0.938	0.938	0.929	0.778
hsa03020	0.00E+00	0.985	0.999	0.966	0.973	0.999	0.993	0.997	0.998	0.976	0.887
hsa03022	0.00E+00	0.949	0.974	0.936	0.957	0.974	0.976	0.913	0.917	0.918	0.834
hsa00900	3.42E-12	0.952	0.972	0.991	0.880	0.996	0.992	0.994	0.993	0.989	0.755
hsa04330	0.00E+00	0.734	0.927	0.861	0.772	0.924	0.837	0.868	0.867	0.898	0.681
hsa04020	0.00E+00	0.669	0.852	0.914	0.783	0.902	0.806	0.848	0.854	0.901	0.621

hsa04970	0.00E+00	0.551	0.798	0.820	0.731	0.785	0.682	0.682	0.683	0.673	0.620
hsa04971	0.00E+00	0.698	0.881	0.830	0.804	0.904	0.823	0.834	0.838	0.822	0.729
hsa03430	0.00E+00	0.972	0.996	0.992	0.964	0.998	0.993	0.990	0.991	0.998	0.864
hsa00040	6.11E-15	0.748	0.928	0.934	0.762	0.915	0.902	0.901	0.908	0.866	0.539
hsa00053	1.11E-16	0.873	0.924	0.962	0.870	0.966	0.974	0.951	0.950	0.938	0.570
hsa00500	0.00E+00	0.791	0.971	0.932	0.750	0.945	0.881	0.872	0.882	0.890	0.572
hsa00860	0.00E+00	0.781	0.885	0.928	0.851	0.948	0.926	0.937	0.935	0.939	0.691
hsa00980	0.00E+00	0.800	0.957	0.977	0.782	0.954	0.922	0.957	0.957	0.957	0.487
hsa00982	0.00E+00	0.839	0.946	0.972	0.799	0.970	0.952	0.964	0.963	0.962	0.799
hsa04610	0.00E+00	0.940	0.982	0.883	0.889	0.994	0.961	0.957	0.966	0.982	0.664
hsa04740	0.00E+00	0.929	0.392	0.982	0.962	0.981	0.278	0.931	0.942	0.523	0.323
hsa00511	3.90E-13	0.929	0.936	0.998	0.732	0.991	0.759	0.984	0.992	0.918	0.738
hsa00340	5.78E-11	0.648	0.881	0.957	0.666	0.828	0.650	0.798	0.817	0.735	0.590
hsa00531	2.22E-16	0.984	0.906	0.996	0.870	0.997	0.940	0.991	0.992	0.953	0.529
hsa00120	6.01E-14	0.988	0.999	0.956	0.927	0.998	0.995	0.981	0.985	1.000	0.689
hsa00620	0.00E+00	0.830	0.917	0.940	0.828	0.944	0.929	0.954	0.955	0.938	0.776
hsa03060	2.22E-16	0.893	0.939	0.976	0.947	0.988	0.962	0.964	0.977	0.969	0.844
hsa05020	1.10E-13	0.672	0.880	0.720	0.829	0.913	0.650	0.765	0.784	0.870	0.630
hsa01040	0.00E+00	0.991	0.995	0.975	0.932	0.998	0.995	0.993	0.992	0.995	0.859
hsa04964	1.16E-11	0.751	0.925	0.947	0.807	0.923	0.911	0.945	0.944	0.924	0.659
hsa00260	0.00E+00	0.872	0.959	0.974	0.854	0.977	0.951	0.956	0.955	0.980	0.682
hsa00785	5.91E-03	0.986	0.971	0.932	0.996	0.995	0.994	1.000	1.000	0.961	0.605
hsa04742	0.00E+00	0.904	0.970	0.935	0.781	0.981	0.687	0.955	0.956	0.861	0.538
hsa00512	0.00E+00	0.994	0.997	0.998	0.993	0.999	0.999	0.999	0.999	0.998	0.981
hsa00910	0.00E+00	0.931	0.928	0.973	0.735	0.989	0.789	0.979	0.978	0.951	0.705
hsa00330	0.00E+00	0.822	0.949	0.919	0.853	0.968	0.941	0.927	0.931	0.943	0.733
hsa00534	0.00E+00	0.896	0.882	0.984	0.900	0.970	0.936	0.986	0.989	0.974	0.765
hsa00350	0.00E+00	0.741	0.853	0.943	0.717	0.891	0.799	0.860	0.878	0.849	0.618
hsa00650	0.00E+00	0.943	0.966	0.982	0.923	0.991	0.969	0.972	0.976	0.973	0.689
hsa00480	0.00E+00	0.841	0.917	0.970	0.824	0.971	0.858	0.949	0.951	0.937	0.593
hsa04614	1.17E-13	0.948	0.992	0.985	0.821	0.990	0.921	0.981	0.980	0.942	0.584
hsa04340	0.00E+00	0.847	0.955	0.930	0.828	0.973	0.674	0.939	0.930	0.960	0.749
hsa00250	0.00E+00	0.868	0.938	0.946	0.871	0.972	0.881	0.933	0.931	0.931	0.713
hsa04930	0.00E+00	0.773	0.892	0.853	0.854	0.951	0.870	0.899	0.900	0.938	0.665
hsa00460	3.18E-05	0.889	0.981	0.997	0.843	0.984	0.975	0.991	0.990	0.996	0.857
hsa00630	6.09E-11	0.821	0.888	0.949	0.859	0.908	0.891	0.927	0.936	0.935	0.764
hsa04744	5.55E-16	0.816	0.918	0.928	0.738	0.970	0.808	0.833	0.851	0.834	0.781
hsa05144	0.00E+00	0.782	0.838	0.907	0.859	0.938	0.589	0.882	0.887	0.844	0.544
hsa00020	0.00E+00	0.952	0.995	0.962	0.947	0.997	0.995	0.990	0.989	0.998	0.789
hsa04320	1.15E-10	0.702	0.846	0.957	0.818	0.907	0.765	0.893	0.885	0.732	0.580
hsa00640	0.00E+00	0.930	0.940	0.951	0.890	0.986	0.966	0.978	0.978	0.967	0.785
hsa00472	2.70E-01	1.000	1.000	1.000	1.000	1.000	1.000	1.000	1.000	1.000	1.000
hsa00360	1.69E-10	0.827	0.931	0.981	0.788	0.970	0.867	0.952	0.956	0.958	0.511

hsa04950	1.44E-15	0.837	0.982	0.953	0.851	0.969	0.911	0.974	0.982	0.979	0.762
hsa05143	0.00E+00	0.855	0.815	0.924	0.902	0.956	0.620	0.873	0.874	0.898	0.631
hsa00790	8.03E-08	0.924	0.845	0.965	0.713	0.975	0.815	0.874	0.877	0.858	0.602
hsa00410	7.77E-16	0.900	0.919	0.967	0.820	0.951	0.902	0.931	0.933	0.932	0.682
hsa00770	6.70E-13	0.949	0.982	0.979	0.753	0.991	0.965	0.983	0.985	0.998	0.762
hsa00051	0.00E+00	0.876	0.896	0.947	0.853	0.982	0.858	0.945	0.942	0.897	0.709
hsa00061	1.68E-05	0.987	0.998	0.997	0.832	0.998	0.998	0.985	0.992	0.998	0.854
hsa00030	0.00E+00	0.902	0.975	0.986	0.790	0.986	0.940	0.935	0.942	0.935	0.777
hsa00052	0.00E+00	0.911	0.981	0.954	0.825	0.989	0.914	0.964	0.971	0.968	0.780
hsa04960	2.22E-16	0.664	0.880	0.870	0.790	0.891	0.877	0.875	0.871	0.909	0.728
hsa00290	2.94E-09	0.962	0.988	0.971	0.894	0.995	0.995	0.999	0.999	0.998	0.870
hsa00604	2.14E-12	0.960	0.947	0.986	0.988	0.992	0.987	0.991	0.992	0.995	0.823
hsa00400	1.26E-04	0.984	0.932	0.997	0.943	0.996	0.991	0.985	0.987	0.993	0.705
hsa00524	9.26E-05	0.999	0.999	1.000	0.955	1.000	0.999	1.000	1.000	0.993	0.851
hsa00730	2.23E-02	0.750	0.917	0.992	0.682	0.900	0.873	0.845	0.875	0.989	0.867
hsa00471	6.75E-04	1.000	1.000	0.987	0.974	1.000	1.000	1.000	1.000	1.000	0.822
hsa04122	3.09E-08	0.949	0.997	0.926	0.886	0.995	0.998	0.994	0.992	0.998	0.829
hsa00750	4.87E-04	0.833	0.911	0.997	0.699	0.961	0.855	0.888	0.908	0.966	0.703
hsa00780	3.67E-02	1.000	1.000	1.000	1.000	1.000	1.000	1.000	1.000	1.000	0.999
hsa00072	1.93E-07	0.948	0.978	0.995	0.886	0.993	0.985	0.994	0.989	0.996	0.832
hsa00740	1.23E-09	0.983	0.904	0.981	0.830	0.998	0.734	0.959	0.962	0.837	0.594
